# Metabolic Stress Induced by Quercetin Enhances Dormancy and Persistence in *Staphylococcus aureus*

**DOI:** 10.3390/antibiotics14050424

**Published:** 2025-04-22

**Authors:** Dae-Youn Kim, Tae-Jong Kim

**Affiliations:** Department of Forest Products and Biotechnology, Kookmin University, Seoul 02707, Republic of Korea; dae111500@kookmin.ac.kr

**Keywords:** intracellular ATP depletion, persister cell, quercetin, *Staphylococcus aureus*

## Abstract

**Background/Objectives**: The persistence of *Staphylococcus aureus* poses a significant challenge in clinical treatments because of its ability to withstand antibiotic therapy. This study assessed the role of quercetin in promoting bacterial dormancy and persistence through ATP depletion and revealed its potential impact on antibiotic tolerance. **Methods**: To assess the effects of quercetin on bacterial metabolism and persistence, *S. aureus* cultures were treated with quercetin, and intracellular ATP levels were then measured. The effect of quercetin on persister cell formation was assessed using antibiotic exposure assays, including pre-treatment and post-treatment strategies. **Results**: Quercetin treatment significantly depleted intracellular ATP levels in a dose-dependent manner, suggesting the presence of metabolic stress. This ATP depletion correlated with increased persister cell formation across multiple antibiotic treatments, indicating that quercetin-induced dormancy enhances bacterial persistence. Notably, quercetin pre-treatment further increased persister cell counts, while delayed quercetin administration increased persister cell survival, highlighting the influence of the timing of metabolic stress on persistence outcomes. **Conclusions**: Quercetin promotes bacterial persistence by inducing ATP depletion and metabolic dormancy. Although quercetin’s bactericidal properties may initially impair bacterial growth, its potential to enhance persistence underscores the complexity of its effects. Further research is necessary to determine optimal strategies for harnessing the antimicrobial properties of quercetin while minimizing its persistence-promoting effects.

## 1. Introduction

Chronic and recurrent bacterial infections pose a major challenge in clinical medicine, often requiring prolonged or repeated antibiotic treatments. A key factor contributing to the persistence of these infections is the presence of persister cells—bacterial subpopulations that exhibit high tolerance to antibiotics without genetic resistance [1,2]. Unlike antibiotic-resistant mutants, which grow in the presence of antibiotics due to genetic modifications, persister cells survive by entering a dormant state that makes them tolerant to antibiotics. These persister cells can later resuscitate and repopulate bacterial communities, resulting in infection relapse even after prolonged antibiotic treatment [3].

*Staphylococcus aureus* is a significant pathogen responsible for various infections, including osteomyelitis, endocarditis, and chronic wound- and implant-related infections [4,5]. Its ability to form persister cells contributes to the failure of antibiotic therapies, making it an ideal model organism for studying bacterial persistence mechanisms. Understanding the factors that influence persister cell formation and survival is crucial for the development of new strategies for treating *S. aureus* infections.

Metabolic activity is a key determinant of bacterial persistence. Persister cells are characterized by low metabolic activity, which limits the efficacy of antibiotics that target actively growing bacteria [6]. Adenosine triphosphate (ATP) depletion has been strongly associated with persister cell formation, as reduced intracellular ATP levels trigger a shift to a dormant-like state that enhances antibiotic tolerance [7,8]. Several studies have demonstrated that persister cells maintain lower ATP levels than metabolically active vegetative cells [7,9], highlighting the role of energy metabolism in persistence. However, the effect of external metabolic stressors, particularly bioactive compounds, on persister cell formation remains poorly understood.

Quercetin, a naturally occurring flavonoid found in various fruits and vegetables [10,11], has been widely studied for its antimicrobial and bioactive properties [12,13]. Studies have reported that quercetin inhibits bacterial growth and interferes with cellular metabolism by disrupting ATP synthesis [14,15]. Given the link between ATP depletion and bacterial persistence, quercetin may play an important role in modulating persister cell formation and survival. However, whether quercetin promotes or inhibits persistence remains unclear. Although its antimicrobial effects suggest a potential role in bacterial eradication, its ability to deplete ATP suggests that it could inadvertently enhance bacterial persistence by inducing a dormant state.

In this study, we assessed the effects of quercetin on the formation and survival of *S. aureus* persister cells under antibiotic treatment. Specifically, we determined whether quercetin-induced ATP depletion influences persister cell populations and whether its effects are dependent on the timing of treatment relative to antibiotic exposure. Understanding the impact of quercetin on bacterial persistence will provide valuable insights into the broader relationship between metabolic stress and antibiotic tolerance, potentially informing strategies for more effective infection control.

## 2. Results and Discussion

### 2.1. Effect of Quercetin on Intracellular ATP Levels

**Figure 1:**
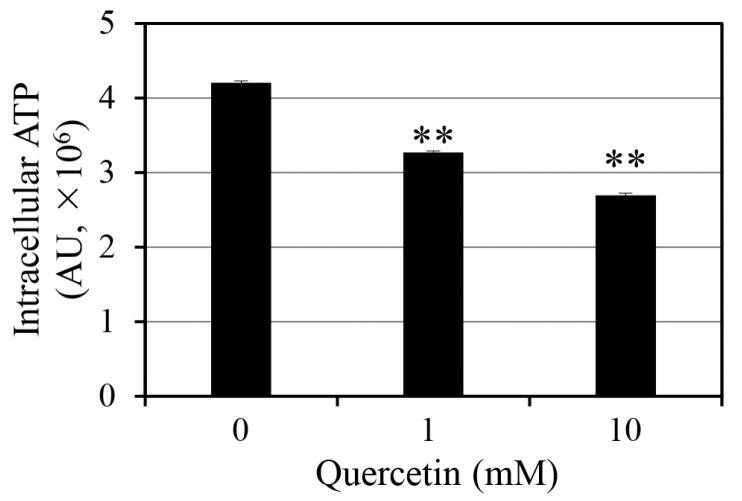
Reduction in intracellular adenosine triphosphate (ATP) levels induced by quercetin dissolved in dimethyl sulfoxide (DMSO). The measured values are expressed as arbitrary units (AU). Data represent means and standard deviations from three independent experiments. Statistical significance was assessed using Student’s *t*-test, with *p*-values < 0.01 indicated by **.

Intracellular ATP depletion is a well-established stress response that promotes bacterial persistence by reducing metabolic activity. To determine whether quercetin induces ATP depletion in *S. aureus*, we measured intracellular ATP levels following quercetin treatment (Figure 1). We noted a significant dose-dependent decrease in intracellular ATP levels, with 1 and 10 mM quercetin reducing intracellular ATP levels by 22% and 36%, respectively. These results indicate that quercetin induces metabolic stress in *S. aureus.*

ATP depletion is a crucial factor in persister cell formation, as it restricts the energy-dependent processes required for antibiotic-mediated killing. Persister cells are characterized by a dormant, metabolically inactive state that enhances their resistance to antibiotics. Quercetin may change this metabolic condition by lowering intracellular ATP levels, thereby creating conditions that favor bacterial dormancy. The results presented in Figure 1 suggest that quercetin increases persister cell formation, based on previous findings that ATP depletion drives bacterial persistence by suppressing energy-intensive processes, such as transcription, translation, and cell wall synthesis [7,9].

The molecular basis for the effect of quercetin on ATP levels appears to be diverse. Quercetin, a polyphenolic compound, disrupts electron transport chain function, thereby impairing oxidative phosphorylation and ATP synthesis in animal cells [16,17]. A previous study has revealed that quercetin interacts with ATP synthase in *S. aureus* [14]. Moreover, the ability of quercetin to generate reactive oxygen species may exacerbate oxidative damage to cellular components in human cells [18,19], suggesting that it further impairs metabolic processes and ATP synthesis in bacteria. This ATP depletion has crucial implications for antibiotic efficacy. Reduced intracellular ATP levels can impair active antibiotic uptake mechanisms, particularly for aminoglycosides, which rely on proton motive force for cellular entry in *Pseudomonas aeruginosa* [20].

### 2.2. Effect of Quercetin on S. aureus Growth

**Figure 2:**
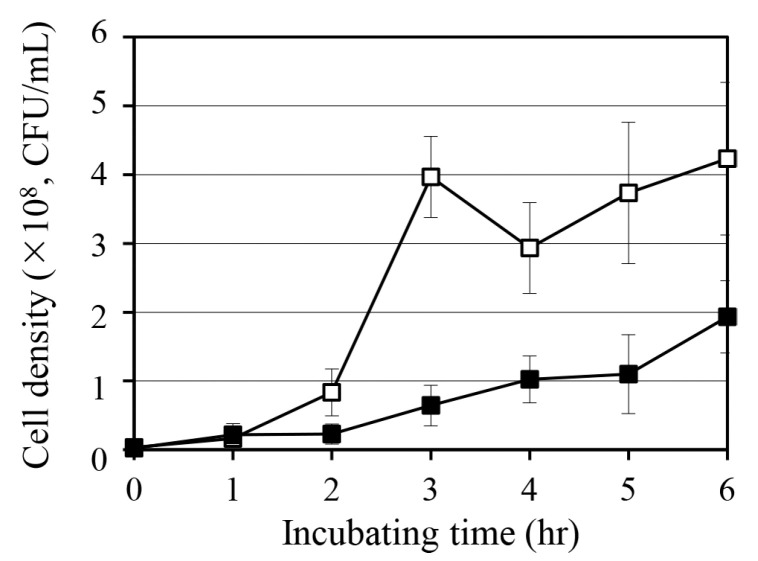
Growth curve of *Staphylococcus aureus* ATCC 6538 in the presence of quercetin. Bacterial growth was monitored in tryptic soy broth (TSB) under two conditions: control (☐, TSB alone) and test (■, TSB supplemented with 10 mM quercetin). Cell density was measured as colony-forming units (CFU). Data represent means and standard deviations from three independent replicates.

To assess whether quercetin induces metabolic stress in *S. aureus*, we monitored bacterial growth following treatment with 10 mM quercetin (Figure 2). Compared with the untreated control, the quercetin-treated group showed significant inhibition of cell growth. In particular, 10 mM quercetin reduced cell density by 54% after 6 h of incubation. This suggests that quercetin acts as a potent stressor that interferes with bacterial proliferation.

This growth-inhibitory effect of quercetin is consistent with its role in interfering with bacterial energy metabolism (Figure 1). A previous study has demonstrated that quercetin reduces intracellular ATP levels in *S. aureus*, possibly by impairing the tricarboxylic acid cycle [21]. By restricting ATP availability, quercetin may compromise essential biosynthetic processes, ultimately reducing bacterial proliferation. As ATP depletion is closely linked to the induction of persister cells, the observed growth suppression may reflect the ability of quercetin to initiate metabolic dormancy.

In addition to ATP depletion, the growth-inhibitory effect of quercetin may be attributed to its deterioration of cell membrane stability. As a hydrophobic compound, quercetin can integrate into bacterial membranes, altering their fluidity and integrity [21]. Membrane disruption can impair nutrient uptake, ion homeostasis, and cellular respiration, all of which contribute to metabolic arrest. This diverse disruption is consistent with the ability of quercetin to inhibit cell growth and suggests that quercetin may promote bacterial persistence.

### 2.3. Effect of Quercetin on Persister Cell Formation

**Figure 3:**
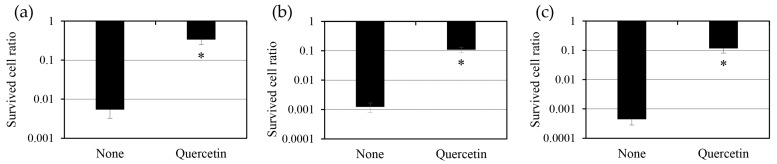
Increase in persister cells of *Staphylococcus aureus* ATCC 6538 induced by 10 mM quercetin. The number of surviving persister cells was determined after treatment with 2.5 mg/mL oxacillin (**a**), 20 mg/mL ciprofloxacin (**b**), and 80 mg/mL tobramycin (**c**) (10× minimum inhibitory concentration). Statistical significance was assessed using Student’s *t*-test, with *p* < 0.05 indicated by *.

To evaluate whether quercetin promotes persister cell formation, we treated *S. aureus* cultures with 10 mM quercetin in combination with three antibiotics targeting distinct cellular processes: oxacillin (a cell wall synthesis inhibitor), ciprofloxacin (a DNA replication inhibitor), and tobramycin (a protein synthesis inhibitor) (Figure 3). Quercetin treatment significantly increased the number of persister cells in all three antibiotic groups. Compared with the antibiotic-only control, we noted a 63-fold increase in the quercetin + oxacillin group, an 88-fold increase in the quercetin + ciprofloxacin group, and a 217-fold increase in the quercetin + tobramycin group. These results strongly suggest that quercetin increases the number of persister cells regardless of the mechanism of action of antibiotics.

The observed increase in persister cell populations may be attributed to the ability of quercetin to induce metabolic dormancy due to metabolic stress. Metabolic dormancy is a well-recognized persistence mechanism characterized by reduced ATP levels, which restrict energy-intensive processes, such as DNA replication, protein synthesis, and cell division—the primary targets of antibiotics. As quercetin reduces intracellular ATP levels, this metabolic shift likely plays a key role in promoting persistence [9]. The correlation between quercetin treatment and increased persister cell formation agrees with previous findings linking ATP depletion to bacterial dormancy and antibiotic tolerance [7,9].

In addition to ATP depletion, quercetin may indirectly enhance persister cell formation by activating bacterial stress response pathways. Under metabolic stress, bacteria commonly initiate the stringent response—a global regulatory system that downregulates growth-related processes while enhancing stress tolerance [22]. By inducing this response, quercetin may increase the expression of genes involved in persister cell formation. Moreover, its membrane-disrupting properties [21] may contribute to increased persistence.

Interestingly, the extent of quercetin-induced persister cell formation varied across antibiotic treatment groups, with the quercetin + tobramycin group showing the most pronounced increase. This variability may reflect differences in antibiotic uptake and intracellular targeting under quercetin-induced metabolic stress. Aminoglycosides like tobramycin rely on an active proton motive force for intracellular entry; therefore, quercetin-mediated ATP depletion may further inhibit tobramycin uptake, enhancing bacterial resistance.

### 2.4. Effect of Quercetin Pre-Treatment on Persister Cells

**Figure 4:**
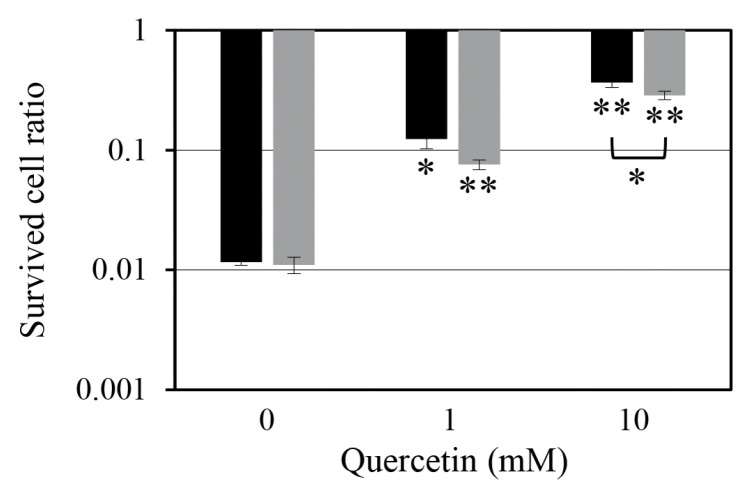
Quercetin-induced concentration-dependent increase in persister cell populations. The effect of quercetin on persister cell formation was evaluated under two treatment conditions: pre-treatment with quercetin 1 h before oxacillin treatment (black bars) and co-treatment with quercetin and oxacillin (gray bars). Cell numbers were quantified as colony-forming units (CFU), and the survival ratio was calculated by comparing the number of persister cells (CFU) after oxacillin treatment with the total number of cells before oxacillin exposure. Data represent means and standard deviations from four independent experiments. Statistical significance was assessed using Student’s *t*-test, with *p* < 0.05 indicated by * and *p* < 0.01 indicated by **.

To determine whether quercetin actively promotes persister cell formation by inducing metabolic stress before antibiotic exposure, we examined the effect of quercetin pre-treatment on persister cell populations (Figure 4). We noted that exposing *S. aureus* cultures to quercetin before antibiotic treatment increased persister cell formation in a concentration-dependent manner. Pre-treatment with 1 mM quercetin for 1 h before oxacillin treatment resulted in a tenfold increase in persister cell populations, while co-treatment with the same concentration of quercetin resulted in a sevenfold increase; however, this difference was not statistically significant. Pre-treatment with a higher concentration of quercetin (10 mM) resulted in a 32-fold increase in persister cell populations, whereas co-treatment resulted in a 26-fold increase. In this case, the increase was statistically significant with *p* < 0.05. These findings highlight that the concentration of quercetin and the timing of its administration play a crucial role in enhancing persister cell formation, with pre-treatment having a more pronounced effect.

The increase in persister cell formation following quercetin pre-treatment re-emphasizes the idea that metabolic stress promotes bacterial persistence. Quercetin-induced ATP depletion may trigger protective metabolic changes before antibiotic exposure, making bacterial cells enter a dormant-like state that is highly resistant to antibiotic-mediated killing. This is consistent with previous findings that metabolic stressors, such as nutrient limitation or oxidative stress, promote bacterial dormancy to enhance survival during antibiotic treatment [23].

Quercetin may also enhance bacterial persistence by activating stress defense pathways. For example, oxidative stress induced by quercetin can activate the SoxRS and OxyR regulons, which enhance bacterial resistance to oxidative damage and contribute to dormancy [24]. By preconditioning cells to withstand oxidative stress and energy depletion, quercetin may increase bacterial survival during subsequent antibiotic exposure. The potential role of quercetin in regulating efflux pump activity should also be considered. Efflux pumps, such as NorA in *S. aureus*, expel toxic compounds, including antibiotics, thereby lowering intracellular antibiotic levels [25]. Many flavonoids inhibit efflux pumps [26], thereby encouraging cells to use alternatives, such as persister cells, to survive antibiotic treatment.

### 2.5. Effect of Quercetin Treatment After Antibiotic Exposure on the Survival of Persister Cells

**Figure 5:**
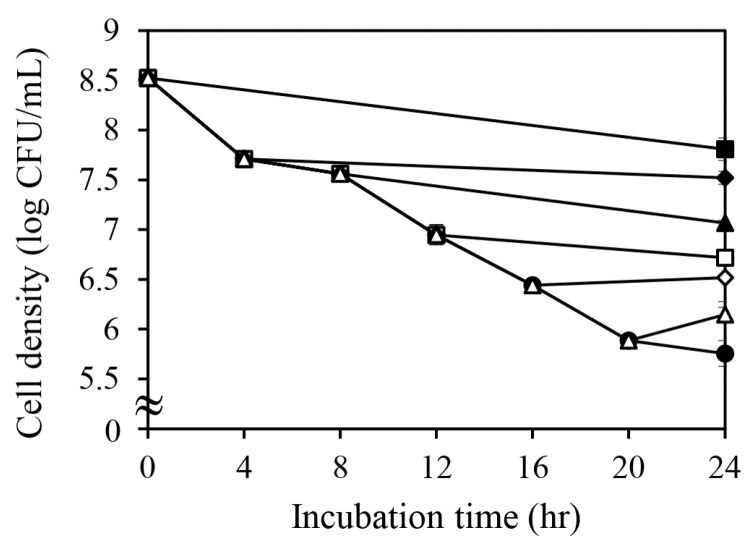
Changes in persister cell numbers following quercetin treatment after oxacillin exposure. Persister cell numbers were assessed at different time points under quercetin treatment after oxacillin exposure (●: no quercetin; ■: 0 h; ♦: 4 h; ▲: 8 h; ☐: 12 h; ◊: 16 h; △: 24 h). Data represent means and standard deviations from three independent experiments.

To determine whether quercetin influences the survival of persister cells following antibiotic exposure, we examined the effect of quercetin treatment after antibiotic exposure on persister cell populations (Figure 5). Quercetin treatment reduced the time-dependent decrease in persister cell populations. The quercetin-treated group maintained a higher number of persister cells than the untreated group. Moreover, faster quercetin treatment could maintain a higher number of persister cells. These results suggest that as quercetin depletes intracellular ATP, the cells do not have enough ATP to restore normal cellular metabolic activity. Therefore, they remain in a dormant state.

The delayed reduction in persister cell numbers supports the hypothesis that ATP availability is crucial for awakening bacterial cells from dormancy and resuming active metabolism. Persister cells, characterized by a low metabolic state, rely on ATP to initiate essential cellular processes, such as protein synthesis, DNA replication, and cell membrane maintenance. Quercetin inhibits these vital processes by limiting ATP recovery in the postantibiotic phase, thereby preventing persister cells from reactivating and becoming susceptible to antibiotic-mediated killing. These findings are consistent with previous findings that ATP levels are a key determinant of bacterial recovery from dormancy [27]. Low ATP levels have been linked to delayed metabolic reactivation and prolonged survival during antibiotic treatment. Cellular reactivation often requires the resumption of energy-producing pathways, such as oxidative phosphorylation. The ability of quercetin to impair these processes through ATP depletion may prolong bacterial dormancy and maintain antibiotic resistance.

The timing of quercetin administration appears to play a crucial role. Administering quercetin immediately after antibiotic treatment may prematurely inhibit ATP recovery, preventing persister cells from reactivating. In contrast, delaying quercetin administration may allow partial ATP recovery, enabling persister cells to become metabolically active and thus vulnerable to antibiotics before quercetin-induced ATP depletion occurs. This suggests that the ability of quercetin to suppress persister cell recovery is time-sensitive and dependent on the balance between ATP depletion and metabolic rebound.

An alternative explanation is that quercetin may disrupt bacterial membrane integrity and nutrient uptake. It may impair membrane function and consequently reduce the influx of essential metabolites required for ATP synthesis, further extending the dormant state. Similar effects have been reported with other polyphenolic compounds that destabilize membrane potential and proton motive force, thereby impairing bacterial energy production [28]. These findings emphasize the complex role of quercetin in regulating bacterial persistence. While the ATP-depleting property of quercetin may interfere with persister cell recovery, this effect could unintentionally prolong the survival of dormant bacteria, potentially increasing the risk of recurrent infection. Understanding these dual effects is essential for designing strategies that can effectively target persister cells while minimizing unintended consequences for bacterial persistence.

Further research is warranted to determine the optimal concentration of quercetin and timing of its administration to maximize its potential for inhibiting persister cell recovery without promoting long-term bacterial survival. Combining quercetin with agents that directly target dormancy mechanisms may enhance its therapeutic efficacy in combating persistent bacterial infections.

### 2.6. Implications of Quercetin-Induced ATP Depletion on Bacterial Persistence

Our findings collectively support the hypothesis that ATP depletion is a central factor in persister cell formation and restoration dynamics. Quercetin-induced ATP depletion not only facilitates the formation of persister cells but also impairs their reactivation following antibiotic exposure, highlighting its dual impact on bacterial persistence.

Previous studies have established that ATP depletion is closely linked to bacterial dormancy, a key feature of persister cells that confers enhanced antibiotic tolerance [7,8,9]. By reducing intracellular ATP levels, quercetin appears to move bacterial cells into a low-energy state, effectively promoting persistence. This mechanism is consistent with previous findings that persister cells maintain suppressed metabolism to survive in hostile environments, including antibiotic exposure [1,2]. Importantly, our results build on these findings by demonstrating that quercetin also delays persister cell recovery, further reinforcing the role of energy restriction in maintaining bacterial dormancy.

However, the paradoxical effect of quercetin has significant clinical implications. While the ability of quercetin to suppress metabolic recovery may offer therapeutic potential for controlling persistent bacterial populations, its ability to simultaneously induce persister cell formation raises concerns about unintended consequences. Quercetin can trigger dormancy mechanisms, inadvertently increasing the likelihood of infection recurrence, particularly if antibiotic treatments fail to eliminate persister cells. This dual role underscores the complexity of targeting bacterial persistence through metabolic interventions.

Moreover, the observed outcomes emphasize the need to explore the broader impact of quercetin on bacterial physiology. In addition to ATP depletion, quercetin may influence other stress response pathways, such as oxidative stress defense mechanisms and toxin–antitoxin systems, both of which are known to contribute to persister cell maintenance. Further studies are warranted to determine whether these secondary effects contribute to the impact of quercetin on bacterial survival strategies.

From a therapeutic perspective, quercetin’s ATP-depleting effect may be beneficial if strategically combined with treatments that exploit bacterial energy depletion. For instance, combining quercetin with antibiotics that rely on active cellular processes for efficacy, e.g., aminoglycosides, may improve treatment outcomes by synchronizing bacterial dormancy with antibiotic susceptibility. On the other hand, combining quercetin with metabolic stimulants that force persister cells out of dormancy may expose these otherwise tolerant cells to antibiotic-mediated killing.

Our findings highlight the importance of evaluating not only the bactericidal effects of metabolic interventions on actively dividing cells but also their potential impact on persister cell dynamics. Future research should focus on identifying conditions that maximize quercetin’s beneficial effects while minimizing its unintended role in promoting persistence. Refining strategies that exploit bacterial energy metabolism can facilitate the development of novel approaches for overcoming antibiotic tolerance and persistent infections.

## 3. Materials and Methods

### 3.1. Strain and Chemicals

*S. aureus* ATCC 6538 was obtained from the Korean Collection for Type Cultures (Jeongeup, Republic of Korea) and stored at −80 °C in a 25% glycerol solution (catalog number: 4066-4400; Daejung Chemical & Metals Co., Ltd., Siheung, Republic of Korea). The bacterial cells were routinely cultured on tryptic soy agar (TSA), which was prepared by adding 1.5% Bacto agar (catalog number: 214010; Becton Dickinson Korea Co., Ltd., Seoul, Republic of Korea) to TSB (catalog number: 211825; Becton Dickinson Korea Co., Ltd., Seoul, Republic of Korea). Liquid cultures were grown in TSB at 37 °C with shaking at 250 rpm.

Quercetin hydrate (catalog number: AC174070250) was purchased from Acros Organics, Thermo Fisher Scientific Korea Ltd. (Seoul, Republic of Korea), and dissolved in dimethyl sulfoxide (DMSO; catalog number: 000D0458; Samjeon Chemicals, Seoul, Republic of Korea) to prepare a stock solution. Ciprofloxacin hydrochloride monohydrate (catalog number: C2227) and tobramycin (catalog number: T2503) were purchased from Tokyo Chemical Industry Co., Ltd. (Tokyo, Japan), while oxacillin sodium salt (catalog number: sc-224180) was purchased from Santa Cruz Biotechnology Inc. (Dallas, TX, USA). All antibiotics were dissolved in distilled water. A saline solution (0.85% sodium chloride [NaCl]) was prepared by dissolving NaCl (catalog number: S0476; Samjeon Chemicals, Seoul, Republic of Korea) in distilled water and sterilized by autoclaving before use.

### 3.2. Measurement of Intracellular ATP Levels

The effect of quercetin on intracellular ATP levels was assessed using the BacTiter-Glo™ Microbial Cell Viability Assay (catalog number: G8230; Promega Korea Ltd., Seoul, Republic of Korea), according to the manufacturer’s instructions. In brief, *S. aureus* was streaked onto TSA plates and incubated at 37 °C for 24 h. A single colony was transferred to a test tube containing 5 mL of TSB and precultured at 37 °C with shaking at 250 rpm for 24 h. In the test group, precultured *S. aureus* was inoculated into a test tube containing 5 mL of TSB at an initial OD_600_ of 0.05 and then incubated for an additional 3 h. The control group was treated with 1% DMSO, while the test group was treated with quercetin. After treatment, 100 µL of the bacterial culture was mixed with 100 µL of BacTiter-Glo™ solution in a 96-well white microplate (catalog number: 30196; SPL Life Sciences Co., Ltd., Pocheon, Republic of Korea). Luminescence was measured using a Synergy™ LX Multi-Mode Reader (BioTek Instruments Korea Ltd., Seoul, Republic of Korea).

### 3.3. Measurement of Growth Curve

The effect of quercetin on *S. aureus* growth was determined by measuring bacterial growth over time. *S. aureus* was precultured under the same conditions as those mentioned above (Section 3.2) and inoculated into a 250 mL baffled flask containing 20 mL of TSB at an initial OD_600_ of 0.05. The control group was treated with 1% DMSO, while the test group was treated with 10 mM quercetin. The cultures were incubated at 37 °C with shaking at 250 rpm for up to 6 h. At every hour, aliquots were collected, washed with sterile saline (centrifuged at 6800× *g* for 1 min), and diluted in saline. Serial dilutions were plated on TSA and incubated at 37 °C for 24 h to determine colony-forming units (CFU). Growth curves were plotted based on CFU from three independent experiments.

### 3.4. Persister Cell Assay

The number of persister cells was evaluated according to a previous study [29]. In brief, *S. aureus* was precultured under the same conditions as those mentioned above (Section 3.2). The precultured strain was then inoculated into 4.5 mL of TSB at an OD_600_ of 0.05 and incubated at 37 °C with shaking at 250 rpm for 3 h. Subsequently, bacterial cultures were treated with the following antibiotics at 10 times the minimum inhibitory concentration: oxacillin (2.5 mg/L), ciprofloxacin (20 mg/L), and tobramycin (80 mg/L). The control group was treated with 1% DMSO, while the test groups were treated with quercetin.

To determine persister cell survival, aliquots were collected at the indicated time in experiments, washed with saline (centrifuged at 6800× *g* for 1 min), and serially diluted in saline. The diluted samples were plated on TSA and incubated at 37 °C for 24 h to determine CFU. The persister cell ratio was calculated as the CFU after antibiotic treatment divided by the CFU before antibiotic exposure. Moreover, the effect of quercetin on persister cell dynamics after antibiotic treatment was examined by adding quercetin at various time points (0, 4, 8, 12, 16, 20, and 24 h) following antibiotic exposure. Three independent experiments were performed for all persister cell assays to ensure reproducibility.

## 4. Conclusions

This study highlights the complex effects of quercetin on bacterial persistence, demonstrating its dual role in promoting the formation of persister cells and inhibiting their metabolic restoration. Although quercetin-induced ATP depletion imposes metabolic stress that enhances persister cell formation, quercetin treatment after antibiotic exposure effectively impairs persister cell recovery by restricting ATP availability during the reactivation phase. These findings underscore the intricate balance between quercetin’s bacteriostatic effects and its potential to unintentionally prolong bacterial dormancy. Understanding this balance is crucial for developing strategies that leverage quercetin’s antimicrobial properties while minimizing its role in persistence. Future research should focus on optimizing timing, dosage, and combination therapies to enhance the therapeutic potential of quercetin in managing persistent bacterial infections.

## Data Availability

The original contributions presented in this study are included in the article. Further inquiries can be directed to the corresponding author.

## Abbreviations

The following abbreviations are used in this manuscript:

AU	Arbitrary units
ATP	Adenosine triphosphate
CFU	Colony-forming units
DMSO	Dimethyl sulfoxide
TSA	Tryptic soy agar
TSB	Tryptic soy broth
